# A Tangle of Curious Forms 

**DOI:** 10.3201/eid3114.AC3114

**Published:** 2025-12

**Authors:** Lesli Mitchell

**Affiliations:** Centers for Disease Control and Prevention, Atlanta, Georgia, USA

**Keywords:** Patrick Mead, fractals, art–science connection, Infection-Associated Chronic Conditions and Illnesses, IACCI

**Figure:**
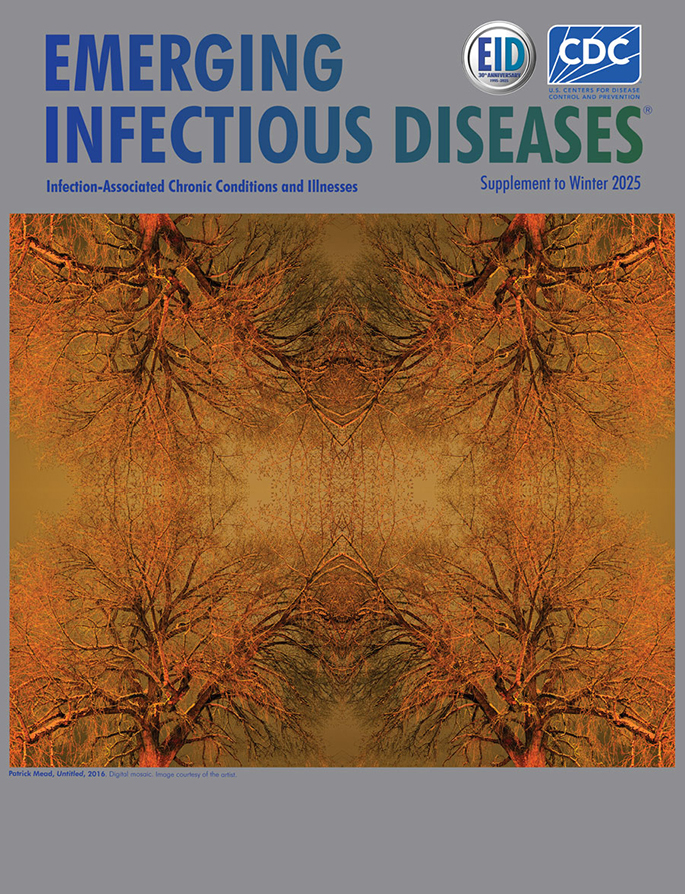
**Patrick Mead, *Untitled*, 2016**. Digital mosaic. Image courtesy of the artist.

I love forms beyond my own / and regret the borders between us

—Loren Eiseley (*1*)

The untitled cover art for this supplement issue of *Emerging Infectious Diseases* was created by Patrick Mead in 2016. Mead’s first experience creating art came at the age of 15, when his parents gave him a digital camera. The artist describes it as his first experience of feeling creative (pers. comm., email, 2025 Dec 9). Initially, his photographic subjects were abandoned structures, such as the Great Western Sugar Company mills found along the Front Range of the Rocky Mountains in Colorado, but his attention turned more toward nature as he grew older.

Mead’s digital image is a mosaic he created in Adobe Photoshop from a single photograph he took of an Eastern cottonwood tree. “The tree was at the intersection of College and Vine in Fort Collins,” said Mead (pers. comm., email, 2026 Jan 8). “I chose it somewhat at random, but also because, like all old cottonwoods, it has this semblance of being a protrusion or extension of the earth beneath it rather than something that rests atop it.”

At the time Mead was creating this piece, he was fascinated by fractal patterns found in the growth of trees and the promise of underlying order and unity that they offered. A fractal is a mathematical equation that describes a complex geometric shape characterized by self-similarity, meaning that it exhibits a similar pattern regardless of scale (*2*). This pattern is also found in nature. A tree is a good example of a fractal pattern: as it grows it repeats the same pattern; each branch has a similar structure to that of the original tree, as illustrated in the Figure.

**Figure:**
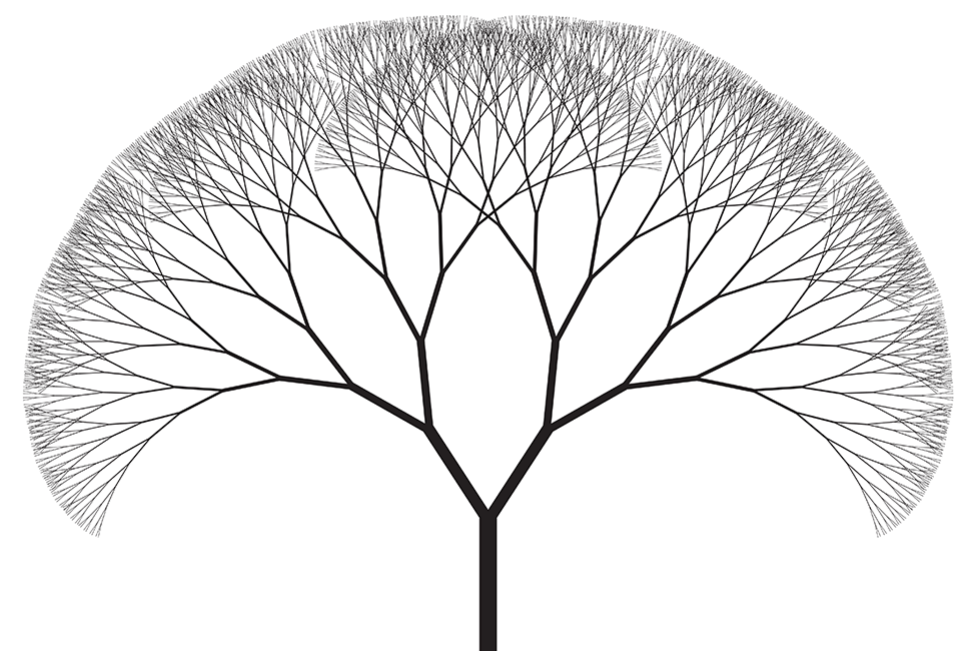
Symmetric fractal binary tree silhouette. Image by synthetick. Source: Adobe Stock (https://stock.adobe.com/1569039416).

Fractals are a newer discovery, the term introduced in 1975 by mathematician Benoît Mandelbrot. Fractals quickly captured the attention of the general public, and the “Mandelbrot set” was featured on the cover of *Scientific American* in 1985 (*3*). Over the course of 50 years, fractals have moved from the realm of mathematics to art and the popular imagination (*4*).

Mead (pers. comm., email, 2025 Dec 9) describes his personal experience of solace in exploring nature’s connectedness:

I found my love for the natural world, of which this image is an early example, grew from my attempts to establish connections with my environment by naming and befriending its nonhuman inhabitants. Isolation can be a pernicious and, at times, self-inflicted condition, independent from one’s social surroundings. Knowing the local flora and fauna proved an effective way for me to combat this. To see the trees you pass every day on your way through the world as fellow inhabitants and companions, friends even, offered me a sense of comfort when it was otherwise lacking.

The series from which this piece originates was inspired by the art of Karl Blossfeldt, a German photographer and key figure in Germany’s Neue Sachlichkeit (New Objectivity) movement, a post–World War I style emphasizing objective, factual representation. Best known for his close-up photography of plants, Blossfeldt, like Mead, was inspired by and spent much time in nature in his youth. Over 30 years, Blossfeldt produced 6,000 photographs using a homemade camera and lens that could magnify up to 30 times (*5*,*6*). The microcosmic aesthetic of his botanical photographs reflected his enduring interest in the repetitive patterns found in nature’s textures and forms, a fascination also shared by Mead. Mead was also inspired at the time the work was created by the writing of Loren Eiseley, a naturalist, anthropologist, writer, and poet. Eiseley’s writings helped inspire the modern environmental movement, and his work explores humanity’s relationship with the natural world.

The artist recognizes that his analysis, while accurate, is one only afforded to him in retrospect. Mead acknowledges that his early interest in the underlying order and unity in patterns found in the growth of trees, and his fuller understanding of the natural world now, are not so different. Mead still practices photography for self-enrichment but says most of his time now is dedicated to his career as an art teacher and to designing board games. He currently resides in Colorado.

This supplemental issue of *Emerging Infectious Diseases* contains articles describing the prolonged, often nonspecific symptoms that arise in some patients after different infections. Collectively, such symptoms fall under the rubric of Infection-Associated Chronic Conditions and Illnesses (IACCIs). In addition to the suffering they cause patients, IACCIs pose a frequent conundrum for healthcare providers. They are familiar in some respects, yet their pathogenesis and full nature are obscure, and their overlap with other diseases fosters misdiagnosis and frustration.

Mead’s cover image is based on reflections and permutations of a single tree. Although the original object is familiar, the viewer’s orientation is upended and confused. What begins as recognizable tree branches overlap and blend into a tangle of curious forms in which different viewers may imagine a host of entities—creatures, faces, capillaries, phantoms—reflecting the multitude of challenges posed by IACCI.
